# Increased decision thresholds enhance information gathering performance in juvenile Obsessive-Compulsive Disorder (OCD)

**DOI:** 10.1371/journal.pcbi.1005440

**Published:** 2017-04-12

**Authors:** Tobias U. Hauser, Michael Moutoussis, Reto Iannaccone, Silvia Brem, Susanne Walitza, Renate Drechsler, Peter Dayan, Raymond J. Dolan

**Affiliations:** 1 Wellcome Trust Centre for Neuroimaging, University College London, London, United Kingdom; 2 Max Planck UCL Centre for Computational Psychiatry and Ageing Research, London, United Kingdom; 3 Department of Child and Adolescent Psychiatry and Psychotherapy, Psychiatric Hospital, University of Zurich, Zürich, Switzerland; 4 Neuroscience Center Zurich, University of Zurich and ETH Zurich, Zurich, Switzerland; 5 Zurich Center for Integrative Human Physiology, University of Zurich, Zurich, Switzerland; 6 Gatsby Computational Neuroscience Unit, University College London, London, United Kingdom; Harvard University, UNITED STATES

## Abstract

Patients with obsessive-compulsive disorder (OCD) can be described as cautious and hesitant, manifesting an excessive indecisiveness that hinders efficient decision making. However, excess caution in decision making may also lead to better performance in specific situations where the cost of extended deliberation is small. We compared 16 juvenile OCD patients with 16 matched healthy controls whilst they performed a sequential information gathering task under different external cost conditions. We found that patients with OCD outperformed healthy controls, winning significantly more points. The groups also differed in the number of draws required prior to committing to a decision, but not in decision accuracy. A novel Bayesian computational model revealed that subjective sampling costs arose as a non-linear function of sampling, closely resembling an escalating urgency signal. Group difference in performance was best explained by a later emergence of these subjective costs in the OCD group, also evident in an increased decision threshold. Our findings present a novel computational model and suggest that enhanced information gathering in OCD can be accounted for by a higher decision threshold arising out of an altered perception of costs that, in some specific contexts, may be advantageous.

## Author summary

Patients with obsessive-compulsive disorder (OCD) report to suffer from indecisiveness and overly cautious decision making. Although many studies captured such a bias experimentally, little is known about the cognitive mechanisms driving such an indecisiveness. In this study, we investigated 16 juvenile OCD patients and compared their performance in a sequential information gathering task to healthy, matched controls. We found an increased information gathering behaviour in OCD. This was accompanied by increased winnings in the OCD group. A newly developed Bayesian computational model revealed that OCD patients outperformed controls in this task because subjective costs for gathering information arose significantly later in the decision making process. This was also reflected by a later collapse in decision boundaries as captured in a delayed urgency signal.

## Introduction

A core feature of psychiatric illness includes personal suffering and functional impairments in daily life [1,2] often coupled with a negative impact on a sufferer’s social environment [3]. Whilst this overall negative impact is well recognised, the possibility that some manifestations of psychopathology might be beneficial is rarely a focus of consideration. Anecdotally, bipolar disorder may be related to creativity [4], while increased exploration in attention-deficit hyperactivity disorder can be beneficial in some limited settings [5,6]. Empirical accounts of the underpinnings of these benefits is sparse, and its deeper understanding could throw light on fundamental aspects of these conditions.

Obsessive-compulsive disorder (OCD) is characterized by intrusive thoughts (obsessions) and/or repetitive behaviours (compulsions) [1,2]. In its first formal definition in the 19^th^ century it was characterised as a disorder of doubt [7,8]. Phenomenologically, patients with OCD show a high level of indecisiveness that impairs efficiency of decision making, even for decisions of little relevance [2,9,10]. An increased intolerance of uncertainty [10–12] and a more cautious decision making style [13–15] are considered to be key features of OCD. For example, in sequential sampling tasks, which allow participants to sample additional information in performing a task, several studies suggest that patients with OCD sample more and are less certain about available options [13,14,16,17], although not unequivocally so [18–20]. Although handicapping in general, it is interesting to conjecture whether these same features might be beneficial in specific contexts, for example where lengthy deliberation carries little cost relative to the cost of a wrong decision.

To examine potential benefits that might arise out of excessive information gathering, and to understand the computational mechanisms underpinning such benefit, we compared performance of a modest sized group of 16 juvenile patients with OCD to that of 16 healthy matched adolescents during performance of a sequential information gathering task. We find that the OCD group outperformed controls in terms of their winnings, an advantage linked to increased information sampling behaviour. To capture the cognitive mechanisms driving this behavioural difference, we developed a novel Bayesian model and show that an elevated decision threshold was the driving factor in patients’ increased information gathering, and this in turn arose out of an altered structure of intrinsic sampling costs.

## Materials and methods

### Ethics statement

The study was approved by the ethics committee of the Canton of Zurich, Switzerland. All participants and their legal guardians provided oral and written informed consent.

### Subjects

Thirty-two adolescent subjects between 13 and 17 years participated in the study. The OCD group consisted of 16 patients recruited from public and private psychiatric practices in the cantons of Zurich, Aarau and Bern (Switzerland). All participants were seeing a clinician due to a primary diagnosis of OCD. Two of the patients were on ward at the time of the study, all others were in outpatient treatment. The controls were recruited from the general population and matched to patients for age and IQ (Table 1). All subjects underwent a structured clinical interview (K-SADS-PL, German version [21]), conducted by experienced clinicians. The OCD group fulfilled the DSM-5 and ICD-10 criteria for OCD at least once in lifetime, and all but one fulfilled the ICD-10 criteria of OCD at the time of the experiment. In addition, self-reported symptom severity in patients was assessed using the CY-BOCS interview [22]. Behavioural results did not change when excluding the subject in remission. Nine patients with OCD were medicated (medication details in S1 Table). No subject from the control group met criteria for major psychiatric disorder based on the clinical interview. A detailed list of comorbidities is provided in Table 1. Some of the subjects participated in an fMRI experiment at a later time point, of which data is reported elsewhere [23]. Participants received vouchers for local stores as reimbursement for their participation (CHF 60). There was no additional reimbursement for actual performance on this task. The study was approved by the ethics committee of the Canton of Zurich, Switzerland. All participants and their legal guardians provided oral and written informed consent.

**Table 1:** Characteristics of the participants.

	controls	OCD	significance
age	15.0y±1.1 (range 13.1–16.8)	15.7±1.5 (range 13.4–17.8)	t(30) = 1.37, p>.05
sex (m/f)	8/8	13/3	χ^2^ (1) = 3.46, p>.05
IQ estimate^1^	113±13	111±25	t(30) = .205, p>.05
medication^2^		SSRI (n = 8) neuroleptic (n = 2)	
symptom severity CY-BOCS (total/obsessions/compulsions)		15.3±9.6 / 7.1±5.3 / 8.2±5.2	
current comorbitities^3^	F40.2 specific phobia (n = 2)	F40.2 specific phobia (n = 2) F90.0 ADHD (n = 2) F91.0 CD (n = 1) F93.8 other childhood emotional disorders (n = 2) F95.1 chronic tic disorder (n = 1)	

Adolescents with OCD were compared to a group of healthy controls that did not differ in their age, IQ or gender. All participants underwent a clinical interview and were screened for medications. ADHD: attention-deficit hyperactivity disorder; CD: conduct disorder; SSRI: selective serotonin reuptake inhibitor. (mean±SD).

^1:^ Waldmann (2008) [78], model 65;

^2:^ Detailed list of medication and doses in S1 Table;

^3:^ Assessed using K-SADS-PL structured interview (German version)

### Task

**Fig 1:**
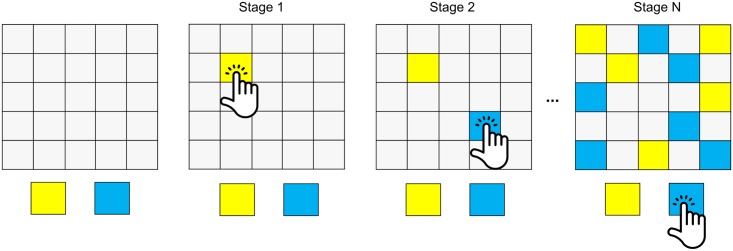
Information gathering task. Subjects have to guess whether the majority of the (initially) hidden cards (left panel) are yellow or blue. They can gather information by selecting cards which then reveal their colour. The subjects are free to open as many cards as they want (middle panels) until they feel certain enough and declare for one colour (right panel). In the ‘fixed’ condition, there is no explicit cost associated with sampling. Correct choices are rewarded with 100 credit points, incorrect ones are punished with the same amount. In the ‘decreasing’ condition, sampling is associated with lower potential wins. For every opened card, subjects will win 10 points less; starting from 250 with no cards opened. False decisions, again, result in a loss of a fixed 100 points.

The participants performed an information gathering task implemented by the CANTAB test system (Fig 1, ‘information sampling task’; Cambridge Cognition, Cambridge UK [18,24]), administered on a touch-screen tablet computer. In each game, subjects were presented with 25 covered cards. They were told each of these cards was either coloured with yellow (*y*) or blue (*b*; sets of colours varied across games, *y* and *b* were chosen here for simplicity). The subjects had to infer on each iteration of the task whether the majority of the 25 cards was yellow or blue. Before declaring their decision as to the majority colour, subjects were free to reveal as many cards as they wished, until they felt certain enough to declare their chosen colour.

The task involved two conditions. The first 10 games belonged to a ‘fixed’ condition, where there was no explicit cost for revealing cards. In this condition, the subject received 100 points upon declaring the correct colour, irrespective of how many cards were opened. A wrong declaration resulted in a loss of 100 points. The second 10 games belonged to a ‘decreasing’ condition, in which subjects could win 250 points for a correct declaration. However, in this iteration turning over of a card led to a reduction in the potential overall winnings amount by 10 points. Thus, if a subject correctly declared after turning 3 cards, then they won 220 points (250–3*10 points). The punishment for a wrong declaration was always 100 points irrespective of the number of cards that had been turned over in that game.

After each game, a waiting period was interposed before subjects continued with the next game. This period was dynamically adjusted to approximately level out differences in timing due to varying response speed in the game. This means that deciding earlier did not lead to the task ending more quickly, i.e. subjects could not increase their reward rate using a fast responding strategy. The colour sequences presented to the subjects were predetermined and were independent of the spatial location of the opened card so that all subjects played with the exact same sequences.

### Behavioural analysis

Based on an hypothesis that patients with OCD may perform better than controls, we first compared the total points won between groups. To further analyse any behavioural differences between groups, we then ran repeated-measures ANOVAs with factor condition (‘fixed’, ‘decreasing’) and group (‘OCD’, ‘controls’), followed by post-hoc t-tests to test specific differences. Although there was no significant group difference in gender (cf. Table 1), there were more males in the OCD than in the control group. To account for any potential confound, we re-ran the behavioural analyses by adding ‘gender’ as a covariate. These analyses did not change any of the results reported below, which means that gender did not impact in any of the reported group differences. To examine whether our task findings were related to self-reported symptom characteristics, we assessed whether indecisiveness or symptom severity in the OCD group, as recorded using the CY-BOCS [22], was related to the behavioural effects found in OCD using Spearman rank correlations. Moreover, we also tested whether medication or a comorbid diagnosis of anxiety (current or lifetime) in OCD patients had any impact on information gathering behaviour.

### Computational modelling

To understand the processes generating any observed behavioural difference between the groups we developed a set of Bayesian generative models, where each model assumed that different characteristics accounted for participants’ behaviour. Models were compared using complexity-adjusted model-fits (AIC and BIC), and the winning model was then used for further analyses. These models are described in full in the supplemental material.

This winning model was based on principles we previously used to model a different sampling task (the ‘Urns’ task) [25]. At its heart is the idea of Bayesian belief formation about the generative probabilities that gave rise to the presented sequences. This belief is coupled to a decision-theoretic choice of action based on inferred subjective costs. At each stage of the game, a subject is assumed to compute long-run state-action or Q-values [26,27] for choosing colour *y*, colour *b*, as well as for continuing sampling (‘not-deciding’, *ND*). The computation of the Q-value for the two colours is based on the probability that the visible evidence was derived from a board favouring the given colour, and the associated rewards/costs of making a correct/incorrect decision. The Q-value for not-deciding was computed as the expected value of the subsequent states, plus a subjective cost per step. The expected values were computed using backward induction based upon solving the Bellman equation [28]. The decision policy was determined using a softmax choice rule with an additional lapse rate parameter.

To understand better the structure of the participants’ subjective costs we compared models with two different cost functions. The first model assumed a cost function that grows linearly with the number of samples, i.e. the subjective cost for continuing sampling is the same irrespective of how much one has already sampled. This would mean that the subjective urge to make a decision is stable over time. The alternative model assumes that the subjective costs increase nonlinearly over samples. This means that subjects become more impatient and feel greater urgency to make a decision as more cards are turned over.

### Decision threshold formation

An alternative way to describe the policy arising from our model is via a threshold on the evidence favouring one or the other colour for making a choice. In simple cases of evidence accumulation and optional stopping, such as standard drift diffusion models [29–31], this threshold is fixed. However, recent computational, behavioural and neurophysiological studies have focused on the possibility that the decision threshold decreases over time, associated with increasing urgency [32–38]. Our model attributes such a decreasing decision threshold to two factors: a) an objective component of an approaching horizon, given knowledge that there is only a fixed number of cards, b) an apparent growth in subjective costs per sampling step, incorporating both explicit and implicit costs.

To understand the dynamic decision threshold in our winning model, we performed two different analyses. First, we calculated the predicted decision threshold based on each subject’s model parameters. At each stage, the decision threshold was computed as the indifference point between the Q-value of the best colour and the one for not-deciding (relative to the total evidence). The differences in the decision thresholds were then compared between the groups by performing independent t-tests for each stage, and then run in a cluster-extent permutation test to assess the significance of threshold-differences (height threshold t = 1, 1000 iterations) on the extent of these effects [39,40]. In a second analysis, we showed how actual choice behaviour had a different relationship to evidence in the two groups. To do that, we plotted decisions as a function of both samples and evidence difference, i.e. to illustrate how much evidence an agent needs for every given stage to make a decision [33]. However, due to few data points in this sample (10 per subject and condition), we could not perform such an analysis with the raw behaviour. We exploited our computational model by taking each subject’s best fitting parameters and then allowing 1000 simulated agents to perform the task. Based on the multitude of generated behaviours, we could then calculate the mean evidence difference for each stage and plot this as a behavioural decision threshold. We again compared the two groups using t-tests and cluster permutation tests to correct for multiple comparison [39,40].

### Model parameter comparison

To understand the aspects of the model that drive the decision threshold and behavioural differences, we compared the model parameters between the two groups using non-parametric Wilcoxon rank-sum tests and corrected for multiple comparisons using Bonferroni correction. A detailed description of model parameter estimation is provided in the supplement.

## Results

### Increased performance in OCD

The OCD group won significantly more points than controls throughout the task (OCD: 1929±268 points, controls: 1406±502, t(30) = 3.67, p = 0.001, mean difference: 522.5, 95%-confidence interval: 231–813). When analyzing the number of points won in the two conditions separately, a repeated-measures ANOVA confirmed a difference in the main effect of group (F(1,30) = 13.48, p = .001, marginal means: OCD: 964, CI: 861–1067, controls: 703, CI: 600–806). Moreover, there was also a main effect of condition (F(1,30) = 7.78, p = .009), but no interaction (F(1,30) = 2.32, p = .138). Post-hoc t-tests revealed the group difference was primarily driven by the decreasing condition (decreasing condition: OCD: 1104±182, controls: 744±227, t(30) = 4.94, p<0.001, mean difference: 360, C.I.: 211–509; fixed condition: OCD: 825±229, controls: 662±398, t(30) = 1.41, p = .168, mean difference: 114, C.I.: -72-397; Fig 2A), indicating OCD patients were more successful in this task in terms of points won.

**Fig 2:**
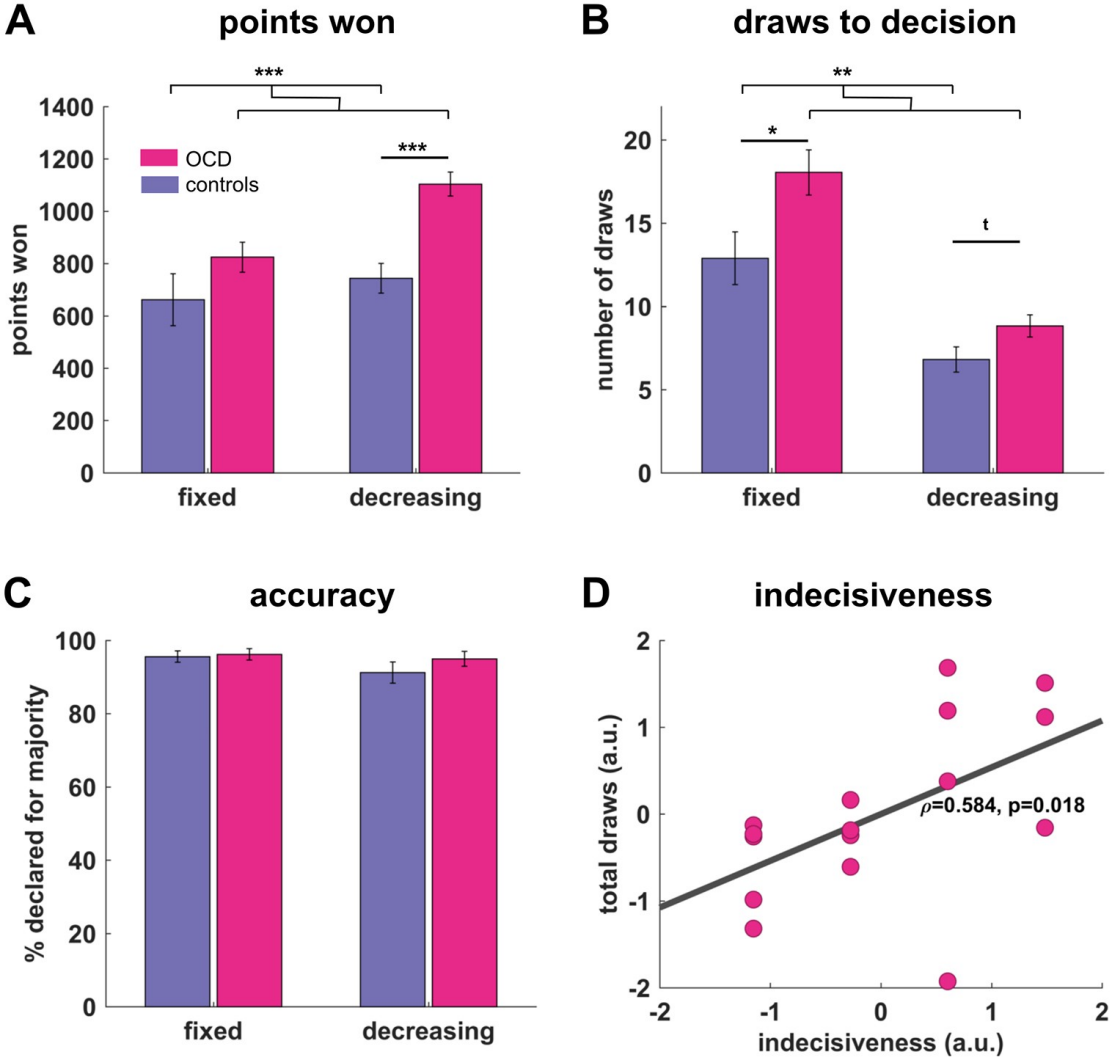
Performance differences in information gathering. Adolescents with OCD won significantly more points than controls across the entire task. This was mainly driven by an increase in points won during the decreasing condition (A). OCD patients opened significantly more boxes (B), but did not differ in performance accuracy (C; accuracy defined as the proportion of choices of the colour that was in the majority of currently opened cards). (D) Total draws to decision correlated significantly with self-reported general indecisiveness in OCD patients, linking task behavior to real-world perceived difficulties. *** p< = .001, ** p < .01, * p < .05, t p < .10.

### OCD patients gather more information

To obtain a deeper understanding of this superior performance we analyzed the number of draws before making a decision and the accuracy of their decisions. OCD subjects turned over significantly more cards compared to controls, as revealed in a main effect of group (F(1,30) = 8.3, p = .007, marginal means: OCD: 13.4, CI: 11.6–15.2, controls: 9.9, CI: 8.1–11.6, Fig 2B). There was also a main effect of task condition (F(1,30) = 51.64, p < .001), but no interaction (F(1,30) = 2.17, p = .151). Post-hoc t-tests show that increased sampling in OCD was apparent in both conditions, but only significantly so in the fixed condition (fixed condition: OCD: 18.1±5.4, controls: 12.9±6.3, t(30) = -2.47, p = .019, mean difference 5.16, CI: .90–9.41; decreasing condition: OCD: 8.8±2.7, controls: 6.8±3.0, t(30) = -2.00, p = .054, mean difference 2.02, CI: -.04–4.08).

### No significant differences in decision accuracy

We analysed participants’ accuracy by comparing how often a subject chose the colour that was more plentiful at the time of decision. There was no significant difference between groups (F(1,30) = 1.2, p = .288, marginal means: OCD: 95.6, CI: 92.7–98.5, controls: 93.4, CI: 90.5–96.4), no condition effect (F(1,30) = 1.7, p = .197) and no interaction (F(1,30) = .54, p = .470). This suggests that neither group was more random at the point of declaring.

### Sequence-dependent performance

To gain further insight into the mechanism accounting for greater winnings in patients, (win more in the decreasing condition, but sample more in the fixed condition), we analysed the sequences’ win probabilities as a function of stage. We found that the sequences were less likely to result in a win around stage 5 (cf. supplementary material for detailed analysis; S4 and S5 Figs). To understand whether lower wins in controls were specifically due to this trough, we simulated balanced sequences and found that the superior performance of patients with OCD was not an artefact of the sequences. In fact the effect remained in other sequences and was best explained by patients’ increased sampling. It is noteworthy that the simulated agents’ increased wins were more prominent in the fixed than in the decreasing condition, in line with our hypothesis of a superior performance of compulsive subjects when the cost of sampling is low.

### Information gathering relates to indecisiveness, but not symptom severity in OCD

The finding of an increased information gathering in OCD raises the question as to whether there is a relationship to behavioural patterns beyond a laboratory task. A general indecisiveness is often reported in OCD as assessed in a clinical interview (CY-BOCS; [22]), but is not taken into account in providing a description of symptom severity. We correlated information gathering behaviour in our task with this self-reported indecisiveness in the OCD group and found a strong correlation with a total number of draws across both conditions (Fig 2D, ρ = .584, p = .018). This was mainly due to the increased sampling in the fixed condition (ρ = .498, p = .049), but was also evident in the decreasing condition (ρ = .441, p = .087). Within the OCD group, we did not observe any relationship of OCD symptom severity with either total points won (CY-BOCS total: ρ = .143, p = .598; obsessions: ρ = .007, p = .980; compulsions: ρ = .143, p = .597) or draws to decision (CY-BOCS total: ρ = .157, p = .563; obsessions: ρ = .348, p = .186; compulsions: ρ = -.044, p = .870). These findings suggest that an increased information gathering is closely linked to self-reported indecisiveness, but not to a symptom severity, among OCD patients. Draws to decision might conceivably reflect a decision trait inherent to OCD, rather than an indication of illness severity. However, the absence of a correlation with symptom severity could also be caused by an imprecise estimate of the OCD severity due to a lack in disorder insight in juvenile OCD [41], or the modest size of our patient group that might not have the sensitivity to detect more subtle associations.

### No effect of medication or anxiety

Because many of our OCD patients received medications, we tested whether behavioural differences (draws to decision, total points won) were equally distributed across medicated and unmedicated patients. We found no significant differences for any of these variables (all p’s > .3). Likewise, a comorbid current or lifetime diagnosis of an anxiety disorder did not affect patients’ behaviour (p’s > .05).

This also held true when we adopted a machine learning approach (5-fold cross-validation regression, cf supplemental information) to evaluate whether an additional variable such as anxiety or medication would improve the prediction of the behavioural markers (draws to decision fixed condition, total points won), over and above a mere OCD diagnosis. While the group regressor predicted both behavioural markers (p’s < .05), neither medication status (p’s>.2) nor anxiety (current and lifetime; p’s>.1) improved the classification. However, given the relatively small patient sample, a replication of these effects is desirable.

### Bayesian computational modelling

To understand behavioural differences at a deeper level, we developed several computational models of the task and compared their performance. The best model was then used to analyse model parameters and decision thresholds further.

We used a two-part process in our model selection. In the first part, we compared three candidate models that embodied different premises. The winning model, ‘M_generative_’ calculates the value for choosing yellow, blue, or continuing sampling. These action values are computed based on an agent’s belief as to whether the sequence-generator is more likely to deal cards of yellow or blue colour (i.e. ‘what generative process is likely to cause this sequence’). The second model, ‘M_majority_’, estimates the action values in the same way as M_generative_, except for assessing a belief as to whether colour yellow (or blue) is more plentiful across all 25 cards in the particular set of cards presented. In fact this latter model implements what participants were instructed to do (i.e. ‘whether there are more cards of yellow or blue’). The third is a heuristic model (‘M_heuristic_’) that involves a simple stopping rule but does not consider the accumulated evidence. Model comparison showed (S1A Fig) that subjects’ behaviour is best reflected by the M_generative_ model.

**Fig 3:**
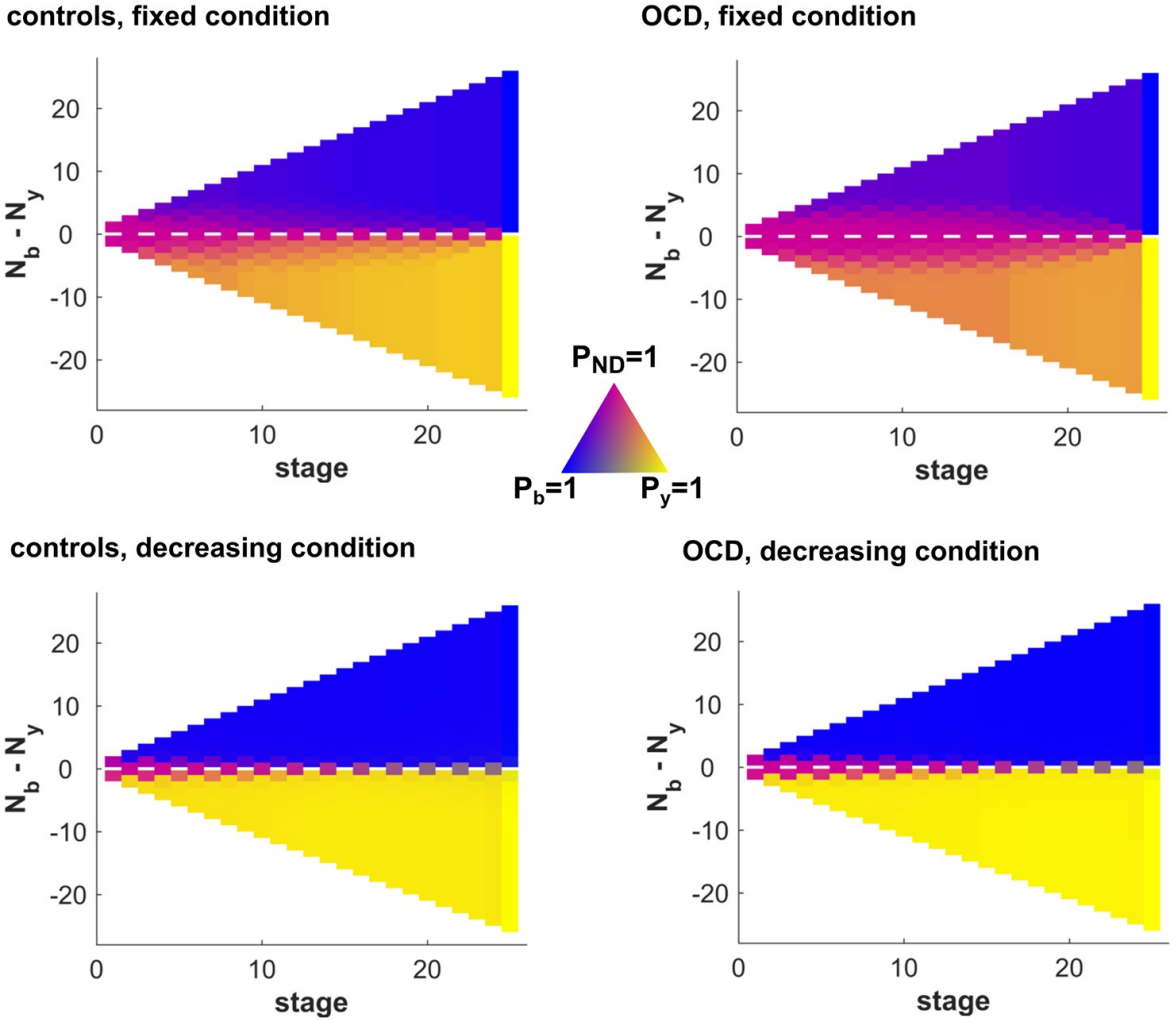
Choice probabilities (policy π) of the winning model. For each stage of a game (x axis), the model predicts the average probability of choosing yellow colour (*P*_*y*_), blue colour (*P*_*b*_), or continuing to sample (pink, *P*_*ND*_). For the fixed condition (top), OCD patients (right) are more likely to continue sampling in conditions where the majority is not clearly apparent. In the decreasing condition (bottom), the probability of not deciding is lower than in the fixed condition in both groups (less pinkish colours). It is also noteworthy that OCD adolescents flexibly adapt their strategy in the decreasing condition, indicating against a general inflexibility in OCD.

We used the winning model from the first part of our model selection (M_generative_; S1B Fig) to compare in the second part whether a linear (as used in part 1) or a nonlinear cost function performed better, and whether free parameters for the cost-functions were the same across both conditions. The final winning model had a nonlinear (sigmoidal) cost function (S2 Fig), defined by three different parameters: a cost parameter *c* describing how costly sampling is in general (i.e. scaling factor); the patience parameter *p* which describes the stage at which a subject becomes impatient, i.e. at what time point the costs start to escalate (i.e. indifference point); the slope parameter *k* describing how quickly a subjects becomes impatient (i.e. slope of the cost function). In the winning model, both *c* and *k* were shared across both conditions, whereas *p* differed for the decreasing and fixed condition. This model outperformed a model where the explicit costs per step (winning 10 points less at every step) in the decreasing condition were modelled in addition to the cost parameters. This suggests that subjects did not take these explicit costs into account accurately. The model predictions (policy) of the winning model are shown in Fig 3.

We also simulated behaviour using the best-fitting parameters for each subject. We found that these simulations produced very similar behaviour to the actual behaviour of our subjects (S3 Fig). Additionally, the model fits did not differ between the two groups, meaning that the model reflected both groups equally well (S3D Fig).

### Elevated decision thresholds in OCD

**Fig 4:**
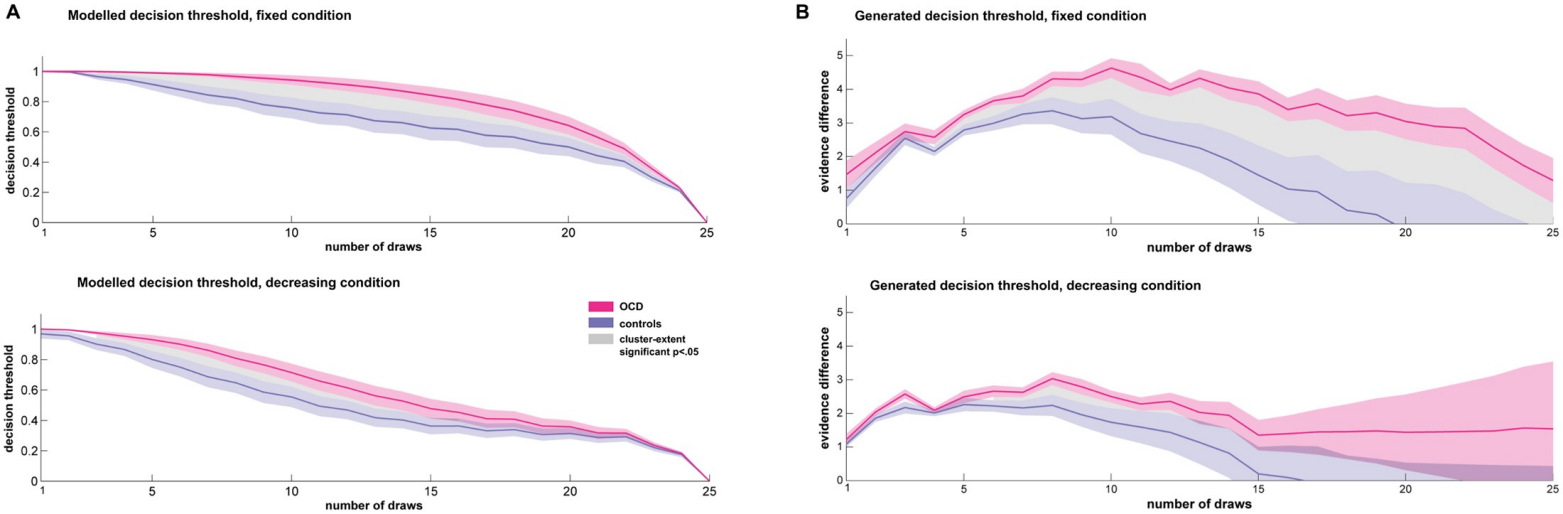
Increased decision thresholds in patients with OCD. The OCD group showed increased decision thresholds in both conditions. The model’s inherent decision threshold showed a distinction between the OCD group and controls (A). When using the model to simulate data, we again found a marked increase in decision threshold (as indicated by the mean evidence difference at choice at each stage) for OCD patients (B). Note the growing evidence difference for the first few draws increases because the magnitude of the difference in evidence is limited by the actual samples (e.g., the difference after 2 draws can be no more than 2). The increasing errorbars in the decreasing condition at later stages shows that even simulated agents rarely sample beyond the 15th step in this condition.

Our model provides an implicit measure of a dynamic decision threshold determining the difference in cards at each stage at which subjects are more likely to declare than to continue sampling. The way these decision thresholds change over samples, and the way they differ between the groups, can reveal about factors such as caution and urgency. We compared the model-predicted decision thresholds at every stage of the game (Fig 4A), and cluster-extent permutation tests revealed an extended increase of the decision threshold in OCD patients in the fixed condition (p = .019) and in the decreasing condition (p = .042).

To verify the effects of an altered decision threshold, we used subjects’ best-fitting parameters to generate simulated data from the model. Each subject’s model played the task 1000 times and we then computed the mean evidence difference for each stage and condition (i.e., evidence difference between the two colours at the stage where the agent chose; Fig 4B). This analysis also revealed an increased decision threshold in the OCD group for the fixed (p = .029) as well as the decreasing condition (p = .013).

### Delayed subjective costs drive increased decision thresholds

To understand how the decision thresholds arose, we compared the model parameters between groups. The main difference was for the patience parameter *p* in the fixed condition (*p*_*1*_: controls: 16.34±7.89, OCD: 22.15±4.80; z(186) = -2.92, p = 0.021, Bonferroni corrected). This difference suggests that the intrinsic costs in patients with OCD arose later than in healthy controls (S2 and S6 Figs). There was also a difference in the slope parameter *k* of the nonlinear cost-function, though this did not survive multiple comparison correction (p = .017, uncorrected; S2 Fig). These findings show that the subjective costs for OCD patients are smaller and escalate later in time, as evident in S6 Fig. This less impatient behaviour in OCD helped them to outperform healthy controls in this task.

## Discussion

Indecisiveness and hesitant behaviour are detrimental to daily functioning, and this is exemplified in the handicaps of patients with OCD, for example their time expenditure on compulsions that impact on social and occupational functioning [42–44]. Here, we analyse the neurocognitive mechanisms underlying an increased information gathering behaviour in juvenile OCD patients, which in turn enabled these patients to win more points than controls.

Our finding of increased information gathering in OCD complements previous studies reporting that patients with OCD tend to sample more in related tasks [13,14,16,17], although the latter observation is not ubiquitous [18–20]. Our computational modelling showed that increased sampling is reflected in a higher decision threshold in OCD patients. The model parameter comparison revealed that this difference is mainly due to lower subjective costs for sampling in OCD patients, reflected in an altered patience parameter. This means that subjective costs, such as impatience, only become important after more samples in OCD. It is critical to note that such subjective costs are only meaningfully defined relative to the potential outcomes of the task. This means that patients with OCD discount subjective costs (which might reflect impatience, fatigue, etc.) more than controls relative to making a correct/incorrect decision. This direct trade-off is also captured by a model comparison where an additional free parameter moderating the subjective impact of being wrong (*R*_*inc*_) failed to improve model predictions. Such a relative discounting of subjective costs may also explain why OCD patients are more indecisive in so far as it allows them to elaborate at greater length before committing to a decision. A close correlation between information gathering and a self-reported, global indecisiveness supports a notion that laboratory elicited increased information gathering has real-life implications. It is interesting to speculate how this might be related to an increased intolerance of uncertainty [10,11] where an intolerance could be a metacognitive consequence of indecisiveness. For example, as patients are aware of their slowness during decision making they might at the same time endeavour to avoid situations that necessitate time-consuming deliberations.

We still know little as to how decision thresholds and altered cost perception are instantiated in the brain. The finding of an urgency gating signal [32–35,45] suggests that decision threshold formation is a dynamic process that changes as function of time. Importantly, such an urgency signal follows a nonlinear increase, similar to that implied in our modelling results [38,46]. While such a signal moderates evidence accumulation in motor and premotor areas [33,38,45], it is unclear where this signal originates. Studies on decision threshold variability hint at an origin in a cortico-striatal loop that includes anterior cingulate cortex (ACC) and subthalamic nucleus (STN) [47–49]. The ACC is reported to show altered function and structure in OCD [23,23,50–54], a target for invasive treatment in OCD [55], while STN is a target for deep-brain stimulation in this condition [56].

It is important to note that an urgency signal in the context of this task, and also in perceptual decision making, is not intended to relate to a concept of ‘negative urgency’ known from addictive behaviours [57–59]. While the latter characterises the tendency to act rashly in negative emotional (distressing) states [57,58], the former is not assumed to be directly related to emotions. Rather, a decision urgency reflects a general property that subjects become more liberal in their decision making as an information sampling process unfolds [32,35]. How such computations link to emotion remains a matter of debate [60]. However, a delayed urgency to decide (as per our model) could reflect an increased urgency to continuing sampling in OCD (i.e. carrying out compulsions) that only slowly wears off as sampling progresses. Such an hypothesis could also be tested in distinct valence contexts, such as a gain vs a loss domain. This could also shed light on an assumed relationship between impulsivity and compulsivity, and between addictive disorders and OCD [61,62].

An advantage of our form of sequential sampling tasks is that all evidence is explicitly displayed on the screen and there is little perceptual uncertainty or working memory capacity limitations that could affect the availability of accumulated information. This is important because facets of how evidence accumulates (such as leakage of evidence) can masquerade as a component of a decision threshold. Thus, our findings complement a recent observation of increased decision thresholds in OCD patients during perceptual decision making [63,64]. An additional benefit of sequential sampling is that it allows us to infer the change in decision thresholds at every stage of the sampling process and investigate the evolution of this threshold over time. Here, a change in decision thresholds was driven by a differential alteration in cost that arises as one continues sampling.

To our knowledge ours is the first study to show such behaviour in juvenile patients with OCD. Previous studies tested adult patients, and most used slightly different tasks, known as ‘Urns’ or ‘Beads’ task [13,14,16,17,19,20]. Only one study used the same information sampling task, but did not find significant differences in draws to decision between the groups [18]. This failure could reflect a variety of factors, such as disorder chronicity in those older patients or effects of long-term treatment. Future studies could usefully compare information gathering in juvenile and adult OCD, or even whether increased information gathering is more closely related to early-onset rather than late-onset OCD subtypes, which might also have different neurobiological aetiologies [65,66]. Such approaches could also help identify potential OCD subtypes that may express an information gathering excess. It would be also interesting to assess whether obsessive-compulsive personality disorder (OCPD) is also associated with increased information gathering, given that indecisiveness and perfectionism are considered as two separate dimensions in OCPD [67], and to assess whether an indecisiveness (as reported in this manuscript) or a perfectionism factor would be more predictive of such behaviour.

Our modelling revealed that the subjective costs did not match the external cost structure in several ways. First, in the fixed condition, where no explicit costs apply, subjects express subjective costs. This is also consistent with the presence of urgency signals in other tasks in the absence of external costs [34,38]. Second, in the decreasing condition, subjects do not take the explicit costs accurately into account, as model comparison revealed. Subjective costs are not represented in a linear manner but as an accelerating function of sampling. Our modelling thus reveals how subjective costs are biased in general and how these give rise to decreasing decision thresholds in particular.

The finding that subjects with OCD outperform healthy controls in our task raises the question as to the ecological circumstances where such advantage might be apparent. One can speculate this might hold in situations where initial uncertainty can be resolved by lengthy in-depth elaboration or where extended elaboration comes at a low cost. However, increased decision thresholds can also be harmful, especially when there is only limited time and many decisions must be made. Previous work has shown healthy humans perform near optimal if they have to trade-off between the time spent for one decision and the number of decisions that can be made in a limited time [68], though this might not be the case for patients with OCD. Moreover, a better performance in juvenile OCD patients could also capture a developmental effect, given that adolescents are assumed to be generally more impulsive [69]. Note we consider extended deliberation times in OCD as active sampling and elaboration, rather than undirected rumination or pondering. One can speculate that an excess of compulsive, repetitive, behaviours and mental rituals in patients is analogous to increased sampling behaviour where the goal is one of attaining full certainty that an intrusive belief will not prove to be real. For example, because an intrusive thought cannot be dismissed with sufficient certainty, patients may develop (irrational) mental rituals that foster an accumulation of additional evidence to attain a decision threshold. The same may apply also to checking behaviour, where one may not trust one’s own actions and continue sampling to cross a high decision threshold.

More recently, an increased in habitual decision making bias has gained much attention in OCD patients and along a compulsivity spectrum [70,71]. Although no study has yet investigated the relationship between habitual behaviour and excessive information gathering, a direct comparison could be informative in understanding the structure of decision making biases in OCD. It is possible altered decision thresholds are directly linked to increased habitual behaviour. In particular, an increased decision threshold during model-based decision making renders it likely that patients with OCD fail to make a decision in the speeded tasks that are used to probe model-based control, especially if we think of this type of reasoning as sampling a state-space (such as Monte Carlo tree search [72]). This means that a model-based system would fail to converge leading to greater reliance on cached, model-free, decision variables that guide habitual decision making. Alternatively, these two decision making biases might be completely independent and thus form two OCD subgroups, one driven by indecisiveness while the other primarily related to an excess in habit formation.

A limitation of our study is the fact that our sample size is relatively small (N = 16) and heterogeneous with respect to comorbidity and medication. To assess the effects of medication on performance, we compared the behavioural (draws to decision, points won) as well as the model parameters between the medicated (N = 9) and unmedicated patients and found no evidence for a significant medication effect. Although previous studies used similar or smaller sample sizes [14,16–18], it is desirable to replicate our behavioural and modelling results in a larger sample. A larger sample could also allow a more detailed assessment of medication effects, potentially informing on neurotransmitter involvement in information gathering and urgency, so extending previous inconclusive studies in relation to the impact of dopamine and ketamine [73–77]. In addition, this could allow further investigation of the effect of symptom severity as well as enable characterization of potential subgroups who might be particularly affected (e.g. checking compulsions). The fact that several patients suffered from comorbidities, such as other anxiety disorders, points to need for validation of our findings in a sample that is matched for other psychiatric dimensions, such as anxiety scores.

### Conclusions

We show that an increased information gathering in juvenile OCD patients is driven by a higher dynamical decision threshold due to a delayed urgency to respond in OCD. In this specific sequential information sampling task more cautious decision making behaviour resulted in higher task winnings in OCD.

## Supporting information

S1 Text Computational modelling of information gathering task. (DOCX)

S1 Fig Model comparison. (A) In the first part, we found that the M_generative_ model (light grey bar) outperformed the other alternative models. This model was then used in part 2 to compare variants of linear and nonlinear cost-functions. The model with a cost-per-step *c* and slope *k* that was shared across conditions, but separate indifference points *p* was the winning model, which was then used for further analysis.
(TIF)

S2 Fig Model parameter comparison. Group comparison of the winning model revealed that OCD had a significantly increased indifference point *p_1_* for the fixed condition. The other parameters did not survive multiple comparison correction. Bottom right: cartoon of nonlinear cost function: *c* moderates the height of the costs whereas *p* determines the change point, and *k* the steepness of the slope. Subscript 1: fixed condition; 2: decreasing condition; ** p = .003, uncorrected.; * p = .017, uncorrected.
(TIF)

S3 Fig Model-generated behaviour. Generated behaviour from the winning model (best-fitting parameters for each subject, running 1000 simulated agents for each subject) produces similar behaviours as found in our groups (Fig 2). The simulated OCD agents win more points (A), make more draws (B), but are similar in their choice acuity (C). (D) The average likelihoods (model performance) for each trial are similar in OCD and controls (z(229) = -1.30, p = .194), meaning that the model performed similar for both groups. (E) Simulated behaviour closely resembles each subjects’ number of draws for both conditions (the closer to diagonal, the more similar).
(TIF)

S4 Fig Sequence-specific performance. (A) The probability of winning changes as a function of stage (black line; mean±s.e.m.): increased sampling leads a higher probability of winning. In the decreasing condition, controls chose at an early stage where the probability of winning was lowest, whereas patients with OCD chose later and thus won more money in this condition. (B) The average number of points that one wins during this task depends on the win probability at that stage, as well as the external costs. Similar to the win probability, the mean points to win has a marked trough around stage 5. It is also visible how this has a bigger impact in the decreasing condition (green), as there are more points at stake during this early phase. It also becomes apparent how the conditions differ in their incentive structure with a vanishing average win in the decreasing condition and an increasing win in the fixed condition (gold). (violet and pink lines indicate choice densities for controls and OCD to indicate the frequency of their decisions as a function of stage).
(TIF)

S5 Fig In silico performance using difference sequences. Simulation of behaviour confirmed our finding that OCD patients outperform healthy controls in terms of their winnings. Even when presented with randomly shuffled sequences, the simulated OCD patients earn more points than the controls (A). In close resemblance of the actual behaviour (Fig 2A–2C), simulated OCD patients made more draws than the controls (B). (C) Shuffled sequences show how win probability increases linearly as a function of stage. ** p < .01; * p < .05; t p < .10.
(TIF)

S6 Fig Lower subjective costs in OCD. The subjective costs increase nonlinearly over time, suggesting that it subjectively becomes more costly to continue sampling as time progresses. For patients with OCD, these subjective costs are less important for their decision making. Especially in the fixed condition (A), OCD patients have a higher patience parameter *p_1_* that indicates that they are more persistent, and less pressed to declare. Please note that the costs per step in the decreasing condition are relative values that are not directly translatable into outcome currency (i.e. number of points) because the outcomes of the winning model do not reflect the actual, objective costs of the task.
(TIF)

S1 Table Medication details. Detailed list of medication usage in OCD patients (one row per medicated patient), listed by subjects receiving medication. N/A: data not available.
(DOCX)
