# Supplementary material for: Increased decision thresholds enhance information gathering performance in juvenile Obsessive-Compulsive Disorder (OCD)
Source: PLoS Comput Biol. 2017 Apr 12;13(4):e1005440. doi: 10.1371/journal.pcbi.1005440 (PMC5406001; doi:10.1371/journal.pcbi.1005440)
Supplement: S1 Text — (DOCX) [file pcbi.1005440.s001.docx]

**Increased decision thresholds enhance information gathering performance in juvenile obsessive-compulsive disorder (OCD)**

TU Hauser, M Moutoussis, R Iannaccone, S Brem, S Walitza, R Drechsler, P Dayan & RJ Dolan

**Supplemental Information**

**Computational models for information gathering**

To better understand the mechanisms during sequential decision making, we developed novel computational models and compared their performance (AIC, BIC) to subsequently study group differences in the best-fitting model. The models M_generative_ and M_majority_ are based on a Bayesian model that we previously established for a related task [25] A third model (M_heuristic_) used an approximate stopping rule, but ignored the magnitude of evidence for a given option. We decided to compare these three models, because they imply different strategies for solving this information gathering task. The M_majority_ model reflects the objective instructions to decide on the majority of cards within the card set. The M_generative_ model is more similar to other tasks, such as the urns task [25], where subjects decide on the generative probability, i.e. what the underlying mechanism is. We also compared these models with a M_heuristic_ model that reflects a simple stopping rule. Additionally, we compared different model versions with varying numbers of free parameters and different cost structures to assess how costs arise as a function of stage.

*Winning M_generative_ model*

We assume that the participants are making inference about the generative process, i.e. the underlying probability causing these draws. This means the agent seeks to make a decision about the probability of the generative process which produced the yellow (*y*) or blue (*b*) cards (*q_y_*, *q_b_*). Its decision for a colour will depend on the probability that the generating process favours that colour over the other one:

$$P(q_{y}>.5|n_{y},N)$$

*n_y_* denotes the number of yellow cards of a total of *N* opened cards at the current stage. This can be expressed as

$$P(q_{y}>.5|n_{y},N)=\int_{q>.5}^{1} P(q|n_{y},N)dq$$

By writing the prior density over *q* as *P*_0_*,* using Bayes rule and marginalizing, we get

$$\begin{matrix} dqP(q|n_{y},N)=\frac{P(n_{y}|q,N)P_{0}(q|N)}{P(n_{y}|N)}dq \\ =\frac{P(n_{y}|q,N)P_{0}(q)}{\int P(n_{y}|q',N)P_{0}(q')dq'}dq \end{matrix}$$

We assume that the probability of *n* yellow draws of *N* total draws follows a binomial distribution and that the prior belief about the generating *q* follows a beta distribution (conjugate prior) with the parameters *α* and *β* (using *α*=1, *β*=1):

$$P(n_{y}|q,N)=\left(\begin{matrix} N \\ n_{y} \end{matrix}\right)q^{n_{y}}(1-q)^{N-n_{y}}=Bin(n_{y}|N,q)$$

$$P_{0}(q)=Beta(q|\alpha,\beta)$$

Thus, the posterior can be expressed as [1]:

$$\begin{matrix} P(q|n_{y},N)dq\propto\\ P(n_{y}|q,N)P_{0}(q)dq=Bin(n_{y}|N,q)Beta(q|\alpha,\beta)dq \\ =Beta(q|\alpha+n_{y},\beta+(N-n_{y}))dq \end{matrix}$$

The beliefs about the generative probabilities are then translated into action-values. The action value of choosing *Y*, (*Q(Y)*), is the product of reward/cost of choosing the right or wrong option (*R_cor_*, *R_inc_*) and the success-probabilities of these actions. *Q(B)* is calculated analogously.

$$Q(Y|n_{y},N)=R_{cor}P(q_{y}>.5|n_{y},N)+R_{inc}P(q_{b}>.5|n_{b},N)$$

The rewards of correctly (*R_cor_*) and incorrectly declaring (*R_inc_*) can be cast in different ways. According to the objective instructions, in the fixed condition *R_cor_* is set to 100 and *R_inc_* to -100. For the decreasing condition, we compared two different formulations. In our main model, we also kept *R_cor_* constant in the decreasing condition. This was done so that the subjective costs (*c_s_*, cf below) soak up the subjectively perceived overall costs, i.e. a combination of externally imposed and internally generated costs. This way, we can investigate the subjectively perceived costs. Alternatively, we formulated an ‘objective’ costs model, where *R_cor_* changes as a function of step (250, 240, 230, …), as set up in the task. The ‘objective’ model only differed in the decreasing, but not in the fixed condition. Additionally, *R_inc_* was kept at -100 for all models and conditions.

The action value of not deciding (*Q(ND)*) computes the value of future states in terms of the future action values and their probabilities. Additionally, a cost per step is imposed that assumes that there are internal (and external) costs that emerge when continuing with sampling. *Q(ND)* is calculated using backward induction to solve the Bellman equation, using state values *V(s’)* and a cost per step *c_s_*:

$$V(s')=\sum_{all available a in s'} \pi(a,s')Q(a,s')$$

$$\begin{matrix} Q(ND|n_{y},N)=c_{s}+\sum_{all s'} P(s'|n_{y},N)V(s') \\ =c_{s}+\sum_{s'=\left\{\begin{matrix} n_{y}+i \\ N+1 \end{matrix}\right\}}^{i=[0,1]} P(s'|n{}_{y},N)V(s') \end{matrix}$$

|  |  |  |
| --- | --- | --- |
|  |  |  |

The probability of reaching state *s’* and seeing *i* new yellow items is based on the current belief status, which in turn is mainly determined by the current evidence *n_y_*, *N*. Thus,

$P(s'|n_{y},N)=P(n_{y}+i,N+1|n_{y},N)$can be calculated as follows:

$$\begin{matrix} P(n_{y}+i,N+1|n_{y},N)=\int_{all q} P(q,n_{y}+i,N+1|n_{y},N)dq \\ =\int_{all q} P(n_{y}+i,N+1|q,n_{y},N)P(q|n_{y},N)dq \\ =\int_{all q} P(n_{y}+i|N+1,q,n_{y},N)P(q|n_{y},N)dq \\ =\int_{all q} P(n_{y}+i|q,n_{y},N)P(q|n_{y},N)dq \\ =\int_{all q} P(i|1,q)P(q|n_{y},N)dq \\ =\int_{all q} Bin(i|1,q)P(q|n_{y},N)dq \end{matrix}$$

The choice policy *π* (cf Fig. 3) for the state-action space is specified as the following softmax function with decision temperature parameter *τ* and irreducible noise (lapse rate) parameter *ξ* [2]:

$$\pi(ND|n_{y},N)=\frac{e^{Q(ND|n_{y},N)/\tau}}{e^{Q(Y|n_{y},N)/\tau}+e^{Q(B|n_{y},N)/\tau}+e^{Q(ND|n_{y},N)/\tau}}(1-\xi)+\frac{\xi}{3}$$

*Alternative M_majority_ model*

As an alternative, we developed a model that the participants are trying to determine the majority of one colour (e.g., yellow: *MY*), rather than just the probability of p>.5 (eq. ).

$$P(MY|n_{y},N)$$

This only has to be calculated when there are less than *N_tot_/2* cards of one colour (i.e. <13) are opened:

$$P(MY|n_{y},N)=\left\{\begin{matrix} if n_{y}>N_{tot}/2;=1 \\ if N-n_{y}>N_{tot}/2;=0 \\ else eq. 1.14 \end{matrix}\right\}$$

where, when the answer is not certain:

$$\begin{matrix} P(MY|n_{y},N)=\int_{0}^{1} P(MY|q,n_{y},N)P(q|n_{y},N)dq \\ =\sum_{Y>N_{tot}/2}^{N_{tot}-(N-n_{y})} \int_{0}^{1} P(Y,N_{tot}|q,n_{y},N)P(q|n_{y},N)dq \\ =\sum_{Y>N_{tot}/2}^{N_{tot}-(N-n_{y})} \int_{0}^{1} P(Y-n_{y},N_{tot}-N|q,n_{y},N)P(q|n_{y},N)dq \\ =\sum_{Y>N_{tot}/2}^{N_{tot}-(N-n_{y})} \int_{0}^{1} Bin(Y-n_{y}|q,N_{tot}-N)P(q|n_{y},N)dq \end{matrix}$$

The first expression is a binomial of getting *Y-n_y_* yellow draws out of *N_tot_-N* draws, given generative probability *q*. The second expression is the probability of the *q* being the generative probability. This can be directly derived from eq. above.

Calculation of *Q(ND)* and the decision policy is similar to the *M_generatinve_* model (eq. ff).

*Heuristic model M_heuristic_*

This model assumes that the participants primarily do not care about ‘how much better’ an option is. Rather, they have an approximate stopping point in mind, i.e. they want to stop at a certain stage and will then select the colour that forms the current majority. This model tests whether subjects actually engage in the task and pay attention to the evidence at hand, or whether they use a simplistic, heuristic strategy.

We will describe the approximate stopping point characterising the agent with the mass density of a unimodal distribution with two parameters characterising the location and width of the peak. We thus raise the binomial mass density *B(N,N_tot_,μ)* to a width-parameter ε, where the policy for deciding for the majority of cards *π(D_maj_| N,N_tot_,μ,ε)* at stage *N* is calculated as:

$$B(N,N_{tot},\mu)=\left(\begin{matrix} N_{tot}-1 \\ N-1 \end{matrix}\right)\mu^{N-1}(1-\mu)^{N_{tot}-N}$$

$$\pi(\mathrm{D}_{maj}|N,N_{tot},\mu,\varepsilon)=\frac{B(N,N_{tot},\mu)^{\varepsilon}}{\sum_{N_{i}=1}^{25} B(N_{i},N_{tot},\mu)^{\varepsilon}}$$

Where $\mu$ describes the peak of the distribution and the probability for choosing the majority at this time point. The policy for non-deciding if the inverse of the choice policy, so $\pi(ND|N)=1-\pi(\mathrm{D}_{maj})$. The parameters for the peak and width of this distribution was fitted for each condition separately.

*Nonlinear sigmoidal cost function*

For the second part of model fitting (cf. below), we compared the winning model of part 1 with a fixed cost per sample to a model in which the costs per sample increased according to a sigmoid function. This could capture the possibility that subjects felt an increasing urgency [32][3] to decide, for instance if it becomes increasingly annoying to gather more samples with potentially little informational content and waste time, similar to previous reports that show that costs increase nonlinearly [38].

We implemented the nonlinear cost function as a sigmoid, where the cost per step *c_s_* (eq. ) changes on each step *n* (1, …, 25):

$$c_{s}=\frac{c}{1+e^{-k(n-p)}}$$

*c* depicts a scaling factor of all costs. The parameter *k* denotes the slope of the increase in costs, whereas the patience parameter *p* describes the indifference point, i.e. at what stage in the game the agent becomes impatient (cf. Fig. S2).

**Model and parameter comparison**

*Methods*

We optimized the models using a genetic algorithm [4] maximizing log likelihood (*LL*). Model comparison was performed using summed AIC [5] and BIC [6]. We used two model comparison parts. We first compared M_generative_ to M_majority_ and M_heuristic_. We then used the winning M_generative_ model and compared different cost implementations during part 2.

To estimate parameters using the genetic algorithm, we used parameter boundaries which were unlikely to be exceeded (e.g. *τ*=[1 50], *p*=[0 25]). For model comparison of the best fitting model, we used an empirical Bayesian approach to regularize and stabilize the parameter estimates. To do so, we created prior distributions for every parameter (beta and gamma distributions) based on the initial parameter fits. Each subject was then re-fitted using these empirical priors, and the resulting parameters were used for parameter comparison. These empirical priors were estimated across both groups and thus likely forcing the final parameter estimates to be more similar between the groups.

*Model comparison results*

Model comparison during part 1 revealed that the M_generative_ model outperformed both the M_majority_ and M_heuristic_ models (Fig. S1A). In part 2, we then compared the winning M_generative_ model based on the standard linear cost function with a model that incorporated the explicit cost structure in the decreasing condition (‘objective’ model). In the latter model, the potential win (*R_cor_*) in the decreasing condition decreases by 10 points at every step, as it is laid out in the task structure. Model comparison revealed that this model performed worse (Fig. S1B). Moreover, analysis of the costs in this model revealed positive costs for the decreasing condition in most subjects (data not shown). This suggests that subjects discount the instructed external costs and that their subjective costs are lower than the externally imposed ones. Subsequently, we compared the linear model to different variants of nonlinear cost functions. We primarily varied the free parameters that were shared between the two conditions. We did so because a standard sigmoidal comes with 3 free parameters (numerator *c*, slope *k*, and indifference point *p*). We found that the best model overall was one that shared one numerator *c* and slope parameter *k* between the conditions, but retained individual indifference points *p* for each condition (Fig. S1B). Additional model comparison reveal that our model had no benefit from an additional free outcome parameter (i.e. potential loss *R_inc_* can deviate from -100). This is because costs-per-step directly trade-off against outcomes (via decision temperatures), and that all biases are directly factorized in the cost term.

**Sequence-specific effects of sampling on winning points**

One revealing statistic is the probability of winning when declaring for the current majority at each stage. To calculate this, we analysed the particular sequences with which subjects were playing and then calculated the probability of winning as a function of stage (Fig. S4A). As expected from the nature of the task, the probability of winning increases as the game progresses. This means that the more a subject samples, the better its estimate of the dominant colour, and the higher the probability to win points.

Given the limited set of sequences, it turns out that the win-probability did not increase monotonically with stage. Rather, it showed a slight trough at stage 5 with a subsequent steep increase. This trough is not only apparent in the win probabilities, but also directly translates in the points that one can win at each stage (Fig. S4B). As it happens, in the decreasing condition, the control subjects had a tendency to declare in that probability-trough (Fig. S4A, violet plot), whereas the patients with OCD enjoyed higher win-probabilities by declaring at a later stage (pink). In the fixed condition, the greater difference in the OCD patients’ propensity to had less impact on the win probability, because both lay in the relatively flat portion of the win-probability curve.

This finding raises the question as to whether it was just unfortunate circumstances (i.e. lower win-probabilities around stage 5) that caused the controls to perform worse. Given that the healthy controls primarily chose around that stage in the decreasing condition, we wanted to test whether this ‘probability-trough’ was responsible for the better performance of the OCD patients or whether the OCD patients would have outperformed the controls also if they were playing with other sequences. To test this, we used our model and the best-fitting parameters (as determined using empirical Bayes) for each subject, playing the task 1000 times and generating behaviours directly from the choice-probabilities of the model. Crucially for this analysis, we used the same sets of card colours, but randomly shuffling their order. This ensured that there was no spurious trough in the win probabilities, but a monotonic increase of the win probability as a function of stage (Fig. S5C).

We then used the simulated behaviour to test whether the groups also differed when playing these altered sequences. Group comparison showed that the simulated OCD group outperformed healthy controls in the decreasing (OCD: 1037.36±315.91, controls: 809.75±270.72, t(30)=2.19, p=0.037; Fig. S5A) as well as in the fixed condition (OCD: 803.71±175.23, controls: 512.49±312.81, t(30)=3.25, p=0.003). Similar to the actual behaviour of our subjects, the simulated OCD patients showed an increased sampling, primarily in the fixed condition (fixed: OCD: 14.45±4.17, controls: 11.04±5.17, t(30)=2.05, p=0.049; decreasing: OCD: 7.94±2.99, controls: 5.98±2.46, t(30)=2.03, p=0.052; Fig. S5B), but no difference in the decision acuity (fixed: OCD: 99.70±0.73, controls: 99.32±0.92, t(30)=1.30, p=0.203; decreasing: OCD: 99.31±1.01, controls: 97.52±4.78, t(30)=1.46, p=0.153). These findings support the notion that the superior performance of the OCD patients is not an artefact of the sequences, but would persist if they faced a difference set of sequences. However, they also highlight that with unbiased sequences, OCD patients would also benefit in the fixed condition in terms of outcomes.

**Classification analysis**

To understand which factors may influence the observed behavioral patterns, we ran a 5-fold cross-validation prediction analysis. We used the task behavior (draws, points won) as a dependent variable in a regression with different independent variables. We then used the regression coefficients from the training sample to predict the task performance on the test sample. This was repeated until we obtained a task prediction for all subjects. To obtain a statistical measure of the predictive validity, we computed the Euclidian distance between the predicted and actual data (L2-norm) and used permutation tests (N=1000 permutations) to obtain a null distribution to define the p-value.

We generally performed two analyses. First, we ran a confirmatory analysis predicting the task scores using a group variable (OCD, controls). This assesses whether we can significantly predict the behavior and is a complementary measure for the frequentists comparisons reported in the main manuscript. To assess whether an additional regressor improved the task prediction (i.e. whether it had a meaningful contribution to task behavior), we extended the independent variables with either anxiety or medication measures and then assessed the performance improvement relative to the simpler model. Permutation tests (permuting the extended regressors) was again used for statistical analysis.

**References**:

1. Bishop C. Pattern Recognition and Machine Learning. New York: Springer; 2007.

2. Guitart-Masip M, Huys QJM, Fuentemilla L, Dayan P, Duzel E, Dolan RJ. Go and no-go learning in reward and punishment: interactions between affect and effect. NeuroImage. 2012;62: 154–166. doi:10.1016/j.neuroimage.2012.04.024

3. Cisek P, Puskas GA, El-Murr S. Decisions in changing conditions: the urgency-gating model. J Neurosci Off J Soc Neurosci. 2009;29: 11560–11571. doi:10.1523/JNEUROSCI.1844-09.2009

4. Goldberg DE. Genetic Algorithms in Search, Optimization, and Machine Learning. 1 edition. Reading, Mass: Addison-Wesley Professional; 1989.

5. Akaike H. Information theory and an extension of the maximum likelihood principle. In: Petrov BN, Csaki F, editors. Second International Symposium on Information Theory. Budapest; 1973. pp. 267–281.

6. Schwarz G. Estimating the Dimension of a Model. Ann Stat. 1978;6: 461–464. doi:10.1214/aos/1176344136
