# Supplementary figures and images for: Increased decision thresholds enhance information gathering performance in juvenile Obsessive-Compulsive Disorder (OCD)
Source: PLoS Comput Biol. 2017 Apr 12;13(4):e1005440. doi: 10.1371/journal.pcbi.1005440 (PMC5406001; doi:10.1371/journal.pcbi.1005440)

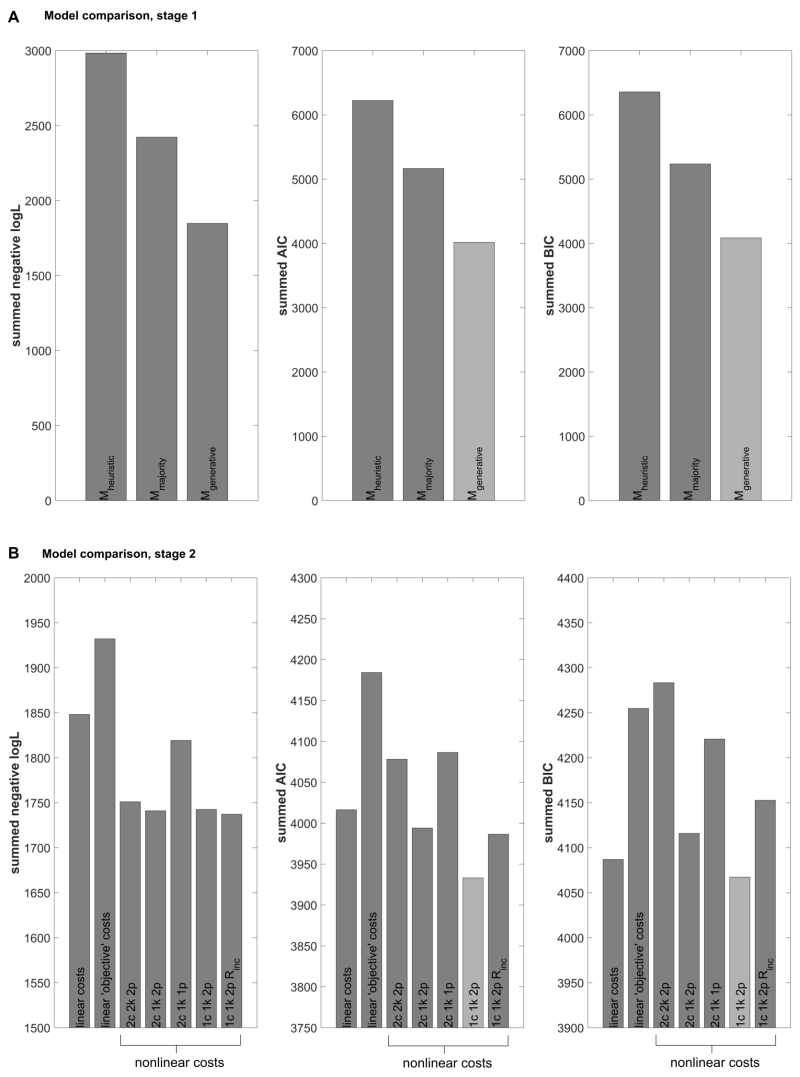

Supplement: S1 Fig — (A) In the first part, we found that the Mgenerative model (light grey bar) outperformed the other alternative models. This model was then used in part 2 to compare variants of linear and nonlinear cost-functions. The model with a cost-per-step c and slope k that was shared across conditions, but separate indifference points p was the winning model, which was then used for further analysis. (TIF) [file pcbi.1005440.s002.tif]

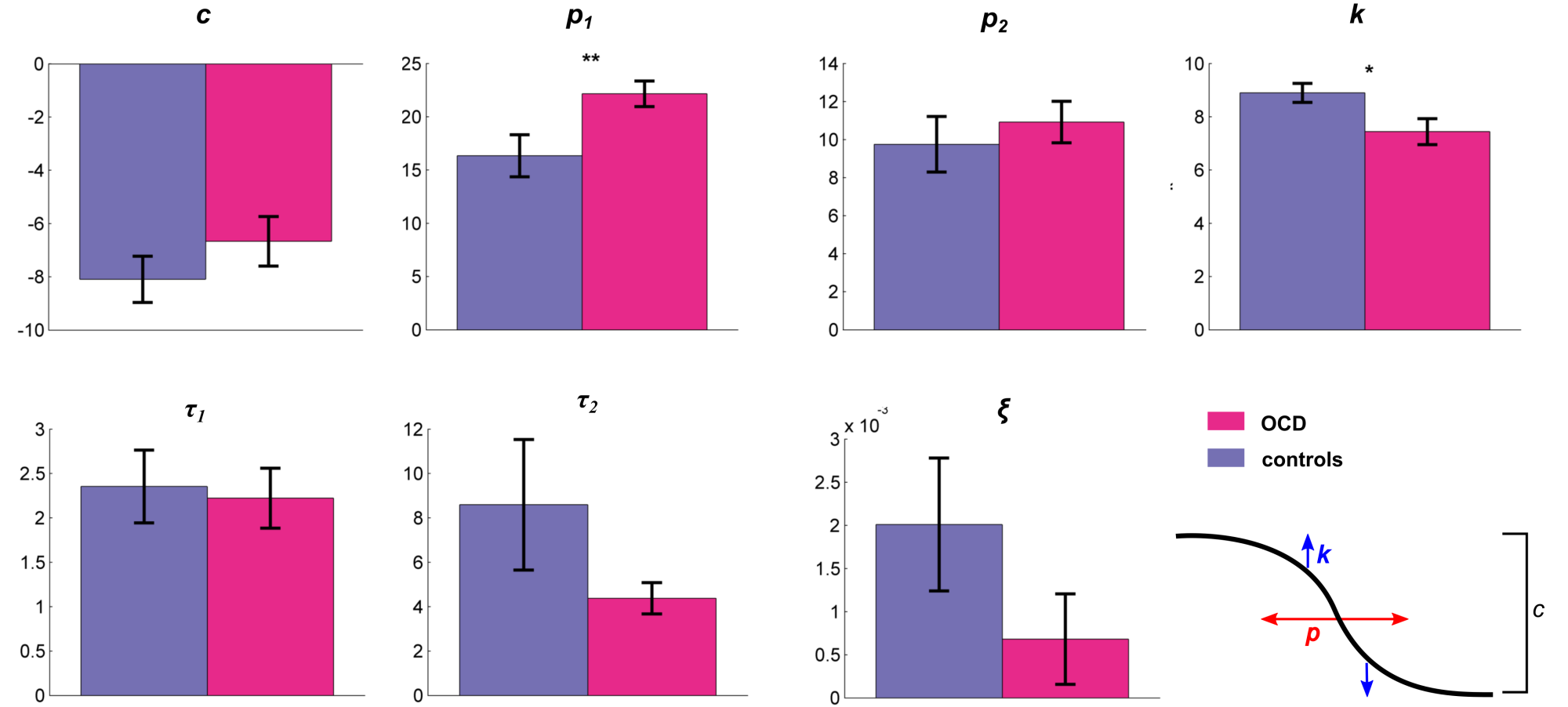

Supplement: S2 Fig — Group comparison of the winning model revealed that OCD had a significantly increased indifference point p1 for the fixed condition. The other parameters did not survive multiple comparison correction. Bottom right: cartoon of nonlinear cost function: c moderates the height of the costs whereas p determines the change point, and k the steepness of the slope. Subscript 1: fixed condition; 2: decreasing condition; ** p = .003, uncorrected.; * p = .017, uncorrected. (TIF) [file pcbi.1005440.s003.tif]

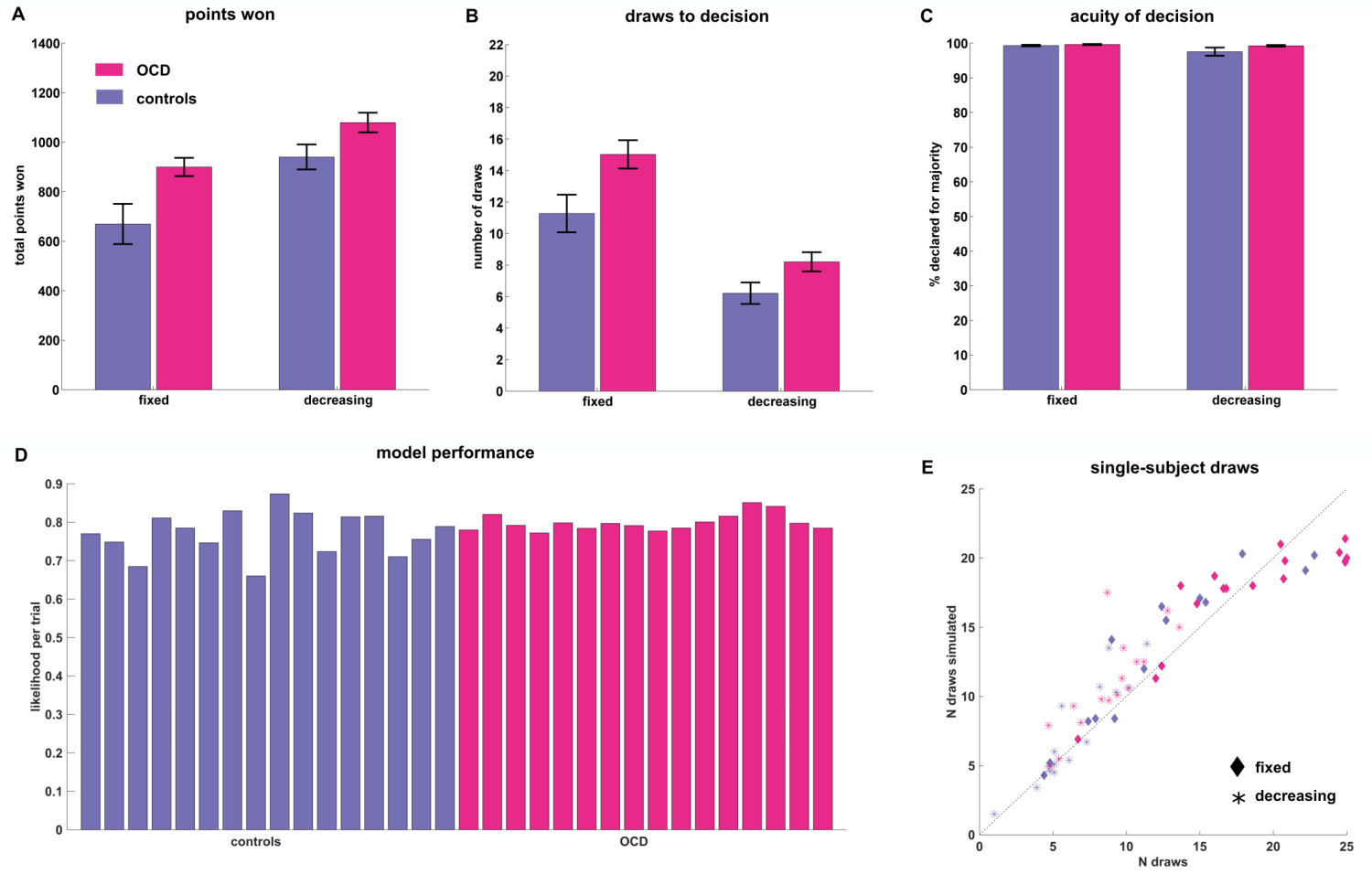

Supplement: S3 Fig — Generated behaviour from the winning model (best-fitting parameters for each subject, running 1000 simulated agents for each subject) produces similar behaviours as found in our groups (Fig 2). The simulated OCD agents win more points (A), make more draws (B), but are similar in their choice acuity (C). (D) The average likelihoods (model performance) for each trial are similar in OCD and controls (z(229) = -1.30, p = .194), meaning that the model performed similar for both groups. (E) Simulated behaviour closely resembles each subjects’ number of draws for both conditions (the closer to diagonal, the more similar). (TIF) [file pcbi.1005440.s004.tif]

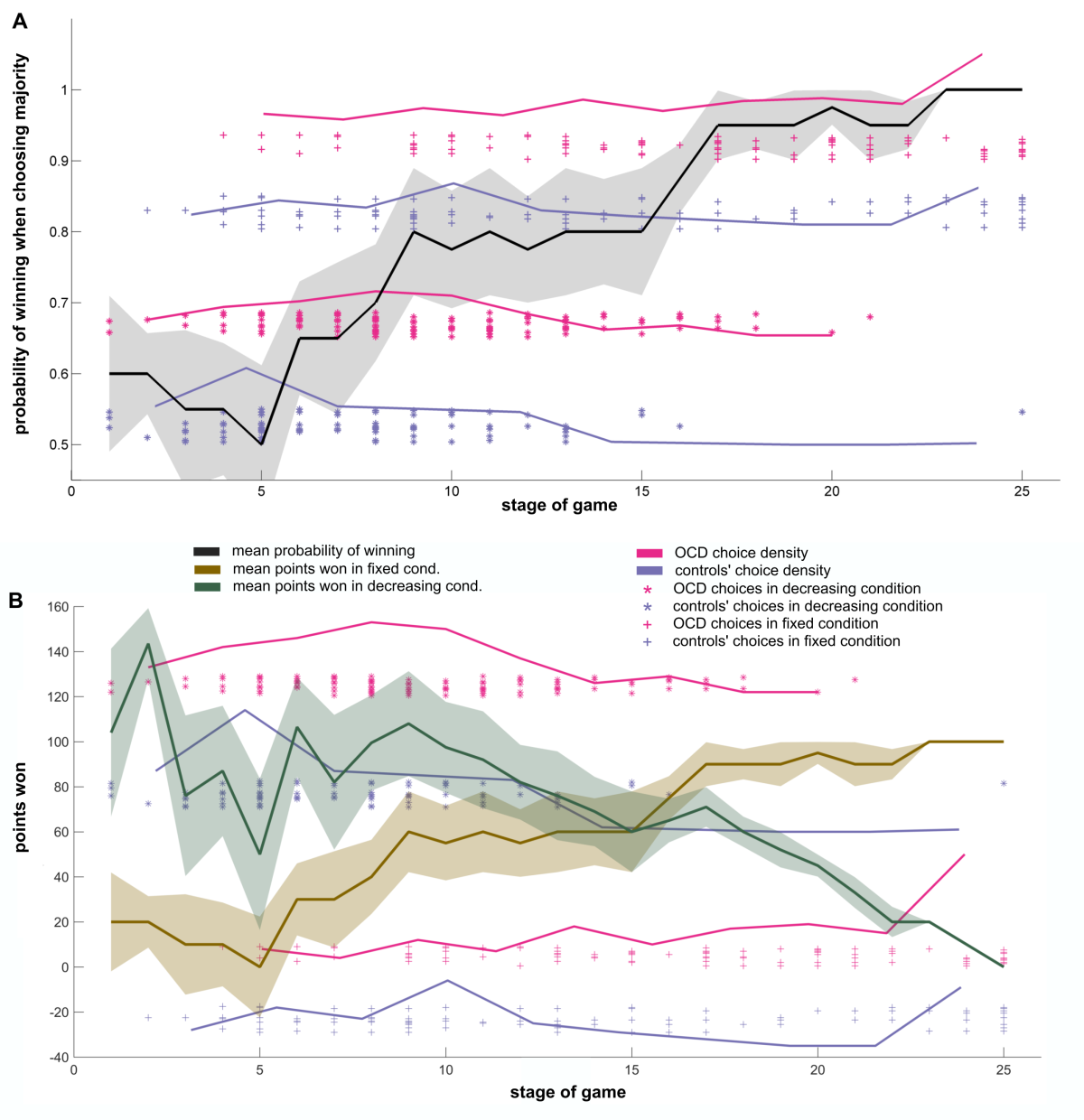

Supplement: S4 Fig — (A) The probability of winning changes as a function of stage (black line; mean±s.e.m.): increased sampling leads a higher probability of winning. In the decreasing condition, controls chose at an early stage where the probability of winning was lowest, whereas patients with OCD chose later and thus won more money in this condition. (B) The average number of points that one wins during this task depends on the win probability at that stage, as well as the external costs. Similar to the win probability, the mean points to win has a marked trough around stage 5. It is also visible how this has a bigger impact in the decreasing condition (green), as there are more points at stake during this early phase. It also becomes apparent how the conditions differ in their incentive structure with a vanishing average win in the decreasing condition and an increasing win in the fixed condition (gold). (violet and pink lines indicate choice densities for controls and OCD to indicate the frequency of their decisions as a function of stage). (TIF) [file pcbi.1005440.s005.tif]

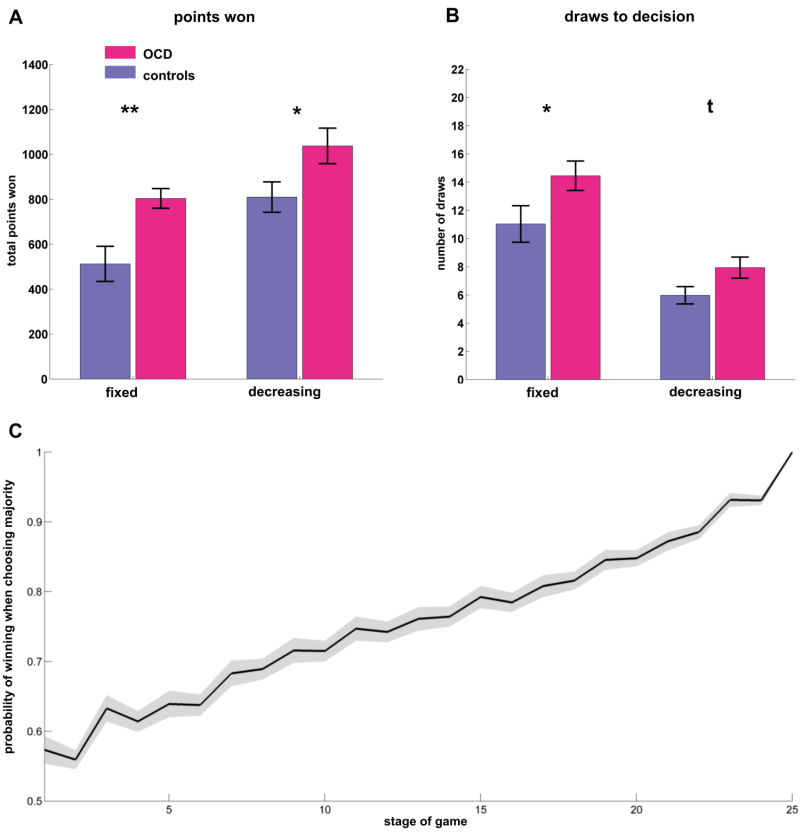

Supplement: S5 Fig — Simulation of behaviour confirmed our finding that OCD patients outperform healthy controls in terms of their winnings. Even when presented with randomly shuffled sequences, the simulated OCD patients earn more points than the controls (A). In close resemblance of the actual behaviour (Fig 2A–2C), simulated OCD patients made more draws than the controls (B). (C) Shuffled sequences show how win probability increases linearly as a function of stage. ** p < .01; * p < .05; t p < .10. (TIF) [file pcbi.1005440.s006.tif]

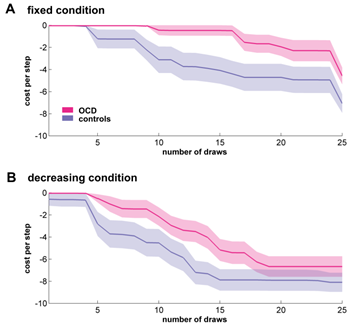

Supplement: S6 Fig — The subjective costs increase nonlinearly over time, suggesting that it subjectively becomes more costly to continue sampling as time progresses. For patients with OCD, these subjective costs are less important for their decision making. Especially in the fixed condition (A), OCD patients have a higher patience parameter p1 that indicates that they are more persistent, and less pressed to declare. Please note that the costs per step in the decreasing condition are relative values that are not directly translatable into outcome currency (i.e. number of points) because the outcomes of the winning model do not reflect the actual, objective costs of the task. (TIF) [file pcbi.1005440.s007.tif]
