# Supplementary material for: Increased decision thresholds enhance information gathering performance in juvenile Obsessive-Compulsive Disorder (OCD)
Source: PLoS Comput Biol. 2017 Apr 12;13(4):e1005440. doi: 10.1371/journal.pcbi.1005440 (PMC5406001; doi:10.1371/journal.pcbi.1005440)
Supplement: S1 Table — Detailed list of medication usage in OCD patients (one row per medicated patient), listed by subjects receiving medication. N/A: data not available. (DOCX) [file pcbi.1005440.s008.docx]

**S1 Table** **Medication details**.

| **drug** | **daily dose** |
| --- | --- |
| Fluoxetine | 10mg |
| Sertraline | 50mg |
| Fluvoxamine | 100mg |
| Sertraline | 150mg |
| Risperidone,  Bipariden | 1mg  2mg |
| Sertraline | 50mg |
| Fluoxetine,  Pipamperon | N/A  N/A |
| Fluvoxamine | 200mg |
| Sertraline | 100mg |

Detailed list of medication usage in OCD patients (one row per medicated patient), listed by subjects receiving medication. N/A: data not available.
